# A Comparative Study of Metaheuristic Feature Selection Algorithms for Respiratory Disease Classification

**DOI:** 10.3390/diagnostics14192244

**Published:** 2024-10-08

**Authors:** Damla Gürkan Kuntalp, Nermin Özcan, Okan Düzyel, Fevzi Yasin Kababulut, Mehmet Kuntalp

**Affiliations:** 1 Department of Electrical and Electronics Engineering, Dokuz Eylül University, İzmir 35160, Türkiye; mehmet.kuntalp@deu.edu.tr; 2 Department of Biomedical Engineering, İskenderun Technical University, İskenderun 31200, Türkiye; nermin.ozcan@iste.edu.tr; 3 Department of Electrical and Electronics Engineering, İzmir Institute of Technology, İzmir 35433, Türkiye; okanduzyel@iyte.edu.tr; 4 Ministry of Transportation, İzmir 35070, Türkiye; fyasink@gmail.com

**Keywords:** metaheuristic, feature selection, respiratory disease classification

## Abstract

The correct diagnosis and early treatment of respiratory diseases can significantly improve the health status of patients, reduce healthcare expenses, and enhance quality of life. Therefore, there has been extensive interest in developing automatic respiratory disease detection systems. Most recent methods for detecting respiratory disease use machine and deep learning algorithms. The success of these machine learning methods depends heavily on the selection of proper features to be used in the classifier. Although metaheuristic-based feature selection methods have been successful in addressing difficulties presented by high-dimensional medical data in various biomedical classification tasks, there is not much research on the utilization of metaheuristic methods in respiratory disease classification. This paper aims to conduct a detailed and comparative analysis of six widely used metaheuristic optimization methods using eight different transfer functions in respiratory disease classification. For this purpose, two different classification cases were examined: binary and multi-class. The findings demonstrate that metaheuristic algorithms using correct transfer functions could effectively reduce data dimensionality while enhancing classification accuracy.

## 1. Introduction

There are many respiratory diseases, such as chronic obstructive pulmonary disease (COPD), asthma, pneumonia, bronchiectasis, bronchiolitis, and upper/lower respiratory tract infections. These diseases are at the top of the list when considering global deaths, emphasizing the importance of their accurate and early diagnosis. Correct diagnosis and early treatment of respiratory diseases can significantly improve the health status of patients, reduce healthcare costs, and improve quality of life. Among the various diagnostic tools available, analysis of respiratory sounds by auscultation is a basic method for identifying respiratory abnormalities. Respiratory sounds such as roughness, coarse crackling, monophonic wheeze, polyphonic wheeze, stridor, bronchus, and squawk provide valuable clues about the respiratory system. Upper respiratory tract infection (URTI), COPD, bronchiectasis, pneumonia, bronchiolitis, asthma, and lower respiratory tract infection (LRTI) are among the most common respiratory diseases that can be detected by auscultation methods. Traditional auscultation relies heavily on the experience and interpretation capability of the physician, which can lead to variabilities in diagnosis. Digital stethoscopes and advanced signal processing algorithms provide a more objective analysis of respiratory sounds. They have also paved the way for the use of automatic decision-making algorithms.

Recent advancements in machine and deep learning algorithms have encouraged researchers working on respiratory sound analysis to develop automated classification systems. Studies in this field fall under two main groups. The first group includes the classification of respiratory diseases, such as asthma, COPD, etc. The second group focuses on classifying respiratory sounds, such as crackle, wheeze, etc. Under these main topics, many valuable studies exist in the literature. Shuvo et al. [1] used a lightweight convolutional neural network (CNN) model, which demonstrates significant efficacy in classifying respiratory auscultation sounds. The model employs a hybrid approach utilizing empirical mode decomposition and continuous wavelet transform, achieving an accuracy of 98.92% in three-class chronic disease classification and 98.70% in six-class pathological classification. Naqvi and Choudhry [2] presented an automated low-cost diagnostic method for COPD and pneumonia, utilizing respiratory sound analysis from the International Conference on Biomedical and Health Informatics (ICBHI) open-access database. The method achieved a classification accuracy of 99.7%. García-Ordás [3] proposed a novel approach utilizing a Variational Convolutional Autoencoder (VAE) combined with a Convolutional Neural Network (CNN) to classify respiratory sounds into healthy, chronic disease, and non-chronic disease categories as well as six specific pathologies. They achieved performance improvements over state-of-the-art methods with a reported F-Score of 0.993 in the ternary classification. Fraiwan et al. [4] investigated the classification of respiratory diseases using respiratory sound signals, achieving an accuracy of 98.27% with boosted decision trees, which outperformed traditional classifiers such as support vector machines. Pham et al. [5] presented a robust deep-learning framework for the analysis of respiratory anomalies and the detection of respiratory diseases using auscultation recordings. They achieved an 84% ICHBI score that averages specificity and sensitivity metrics, which surpasses the previous state-of-the-art result of 72%. In another study by Pham et al. [6], an inception-based deep learning model was developed to detect respiratory anomalies and respiratory diseases from audio recordings, utilizing the ICBHI benchmark dataset. The model achieved 0.53/0.45 ICBHI scores (arithmetic and harmonic averages of sensitivity and specificity) for respiratory anomaly detection and 0.87/0.85 for disease prediction, outperforming several state-of-the-art systems. Kababulut et al. [7] introduced a clinical decision support system for respiratory disease identification using decision tree algorithms and a Shapley-based feature selection to improve performance. Their findings highlight that effective feature selection significantly enhances classification performance in respiratory disease detection. Sfayyih et al. [8] analyzed deep learning applications in respiratory sound analysis, focusing on the effectiveness of CNNs in classifying respiratory sounds. The authors concluded that deep learning techniques show high accuracy in diagnosing respiratory conditions, underscoring AI’s potential in medical diagnostics.

It is widely known that feature extraction has a substantial impact on the efficiency of clinical decision systems. The literature presents numerous diverse feature extraction methods. Classical methods such as Fourier Transform [9], Empirical Mode Decomposition [1], Wavelet Transform [1,6], and Mel-Frequency Cepstral Coefficients (MFCC) [10,11,12,13,14,15] are among the most commonly used methods for feature extraction in the respiratory sound classification field. The proper selection of the most descriptive feature subsets from all extracted features has also been very important for the success of the classification system. Through feature selection, the computational burden on the classifier is reduced by employing a smaller feature set, and in addition classification performance is increased. Therefore, finding effective feature selection (FS) methods has been an extensively studied topic. Filter, wrapper, and embedding techniques are the three general categories into which feature selection methods fall [16]. Kang et al. [17] provide a comprehensive overview of FS techniques, highlighting their significance in managing the challenges posed by high-dimensional datasets. Iqbal et al. [18] presented a comprehensive approach to feature extraction and selection from physiological signals.

In recent years, the application of nature-inspired metaheuristic algorithms for feature selection has gained significant attention within the machine-learning community. Metaheuristic algorithms are primarily used as wrapper-type feature selection methods. Metaheuristic methods range from well-established techniques, like genetic algorithm (GA) and particle swarm optimization (PSO), to newer and more creative approaches, such as the grey wolf optimizer (GWO), teaching learning-based optimization (TLO), Whale Optimization Algorithm (WOA), and the Equilibrium Optimizer (EO). The comprehensive review conducted by Nssibi et al. [19] evaluated various metaheuristic techniques, highlighting their effectiveness in navigating the complex search space associated with feature selection tasks. Sathiyabhama et al. [20] introduced a novel computer-aided diagnosis (CAD) system that employs a GWO and rough set-based approach to identify abnormalities in mammogram images effectively. Kang et al. introduced the Two-Stage Teaching-Learning-Based Optimization (TS-TLBO) algorithm, which demonstrates significant improvements in classification accuracy [17]. Nadimi-Shahraki et al. [21] presented an enhanced version of the Whale Optimization Algorithm (E-WOA) specifically tailored for medical feature selection with a focus on the COVID-19 case study. The experimental results demonstrated that E-WOA significantly outperforms traditional WOA variants and other well-known optimization algorithms. Chen et al. introduced a novel approach that combines particle swarm optimization (PSO) with the 1-nearest neighbor (1-NN) classifier, demonstrating its effectiveness on various life science datasets [22]. Elgamal et al. [23] introduced an enhanced version of the Harris Hawks Optimization (HHO) algorithm, termed Chaotic Harris Hawks Optimization (CHHO), which integrates chaotic maps and simulated annealing (SA) to address the limitations of the standard HHO. Rajammal et al. [24] presented a binary improved grey wolf optimizer (BIGWO) that integrates a mutation operation and an adaptive k-nearest Neighbor (AKNN) algorithm to enhance feature selection efficacy. Prabhakar and Won [25] proposed several innovative techniques, including metaheuristics feature selection methods for classification in telemedicine applications. The study highlights the effectiveness of these methods in analyzing respiratory sounds and showcases the potential for enhanced diagnostic capabilities in healthcare settings. Abedi et al. [26] developed an innovative algorithm that utilizes GA and support vector machine (SVM) classification to analyze thoracic respiratory effort and oximetric signal features. Álvarez et al. [27] conducted a comprehensive study for detecting OSA patients. The authors employed GA for feature selection, achieving very high diagnostic accuracy. All of these studies show that metaheuristic feature selection methods have been successful in addressing the difficulties presented especially by high-dimensional data. Classifier models using features selected by metaheuristic methods enhance prediction accuracy, decrease computing costs, and clarify the process by eliminating unimportant features.

Though successfully used for feature selection in many studies, there is not much research on the utilization of metaheuristic feature selection methods in respiratory disease classification. This study aims to conduct a detailed and comparative analysis of metaheuristic optimization methods in respiratory disease classification. For this purpose, various features were extracted from audio recordings obtained from the publicly available ICBHI 2017 Respiratory Sound Database [28] using 15 frequently used feature extraction techniques. Then, by employing diverse statistical metrics on the collected numerical data, a new feature set was created. Next, to determine the best features that enhance classification performance, six well-known metaheuristics methods were employed with eight transfer functions. Finally, the performances of each method were measured and compared with each other using a simple and identical KNN classifier. In this study, two different classification problems were examined. The first one was a binary classification task (respiratory disease vs healthy) while the second one was a multi-class task (healthy, chronic respiratory disease, nonchronic respiratory disease). Since the database used is highly imbalanced, MCC metric was used as the main performance metrics instead of accuracy. The findings demonstrate that metaheuristic algorithms using correct transfer functions could effectively reduce data dimensionality while enhancing classification accuracy.

This paper is organized as follows: Section 2 provides information about the materials and methods used, including the feature extraction methods from audio recordings, implementation of metaheuristic feature selection methods and transfer functions, classification stage, and evaluation of the results. Section 3 conducts a detailed comparative analysis based on the results obtained. Finally, Section 4 presents the discussion and conclusions of the study.

## 2. Materials and Methods

### 2.1. Data Source

The present study utilizes the openly accessible ICBHI 2017 Respiratory Sound Database [28]. ICBHI 2017 comprises 5.5 h of recordings obtained from seven different chest locations, namely trachea, left and right anterior, posterior, and lateral. The recordings encompass 6898 breathing cycles, labeled by respiratory specialists as containing crackles, wheezes, a combination of both, or no abnormal respiratory sounds. The database was compiled from 126 individual participants over several years by two separate study teams located in two countries. The database contains 920 annotated audio samples from 126 participants (77 adult/49 children and 79 male/46 female), which were recorded using heterogeneous types of equipment, namely Meditron, LittC2SE, Litt3200 stethoscopes, and AKGC417L microphones. The age of the participants is 43.0 ± 32.2 years. Participants included patients with lower respiratory tract infections, upper respiratory tract infections, pneumonia, COPD, asthma, bronchiolitis, bronchiectasis, and cystic fibrosis. The respiratory cycles were also categorized into eight distinct conditions by experts: URTI, Chronic Obstructive Pulmonary Disease (COPD), Bronchiectasis, Pneumonia, Bronchiolitis, Asthma, LRTI, and Healthy.

### 2.2. Feature Extraction

The feature extraction phase was executed utilizing Python’s Librosa library Ver.0.10 [29]. The default value of the library was utilized as the number of features to be extracted for each method. In this study, 15 distinct methods were employed for the feature extraction procedure. The specific characteristics of these methods are displayed in Table 1 [30]. Since the number of features obtained from each method is very large (several thousands), a new and reduced feature set was generated by computing five statistical measures (minimum, maximum, mean, standard deviation, and skewness) of feature values for each feature extraction method. Then, these features were concatenated to form the feature vector for each sample. This way, each sample is represented by 15 × 5 = 75 feature values.

### 2.3. Classification Cases Examined

Once the feature extraction step was completed, feature selection and classification stages followed. This study examines two different cases. Case 1 examines a binary classification task where respiratory sound recordings are categorized into two classes diseased and healthy. The disease class consists of recordings that belong to individuals with seven respiratory diseases specified in Section 2.1; the healthy class comprises data collected from individuals who do not have any respiratory illnesses. Case 2 pertains to a multi-class categorization with three distinct classes: Chronic Respiratory Diseased, Non-chronic Respiratory Diseased, and Healthy. The Chronic Diseased class was formed by amalgamating instances of individuals with COPD, asthma, and bronchiectasis, which are representative of chronic respiratory conditions. The Non-chronic Diseased class consists of respiratory sound recordings obtained from individuals diagnosed with pneumonia, URTI, bronchiolitis, and LRTI. The scenario for the healthy class is analogous to Case 1. Table 2 provides information about the datasets utilized.

### 2.4. Feature Selection by Metaheuristic Methods

This study examines six different metaheuristic methods with various transfer functions to identify the most effective approach for classifying respiratory diseases from respiratory sounds. The methods include genetic algorithm (GA), particle swarm optimization (PSO), grey wolf optimization (GWO), teaching learning-based optimization (TLO), Whale Optimization Algorithm (WOA), and the Equilibrium Optimizer (EO). Each algorithm was executed in 25 trials using identical experimental settings to achieve statistically meaningful outcomes. In each trial, the population was initialized with a size of 100 individuals, and the simulation was run for 50 iterations. 

**Figure 1:**
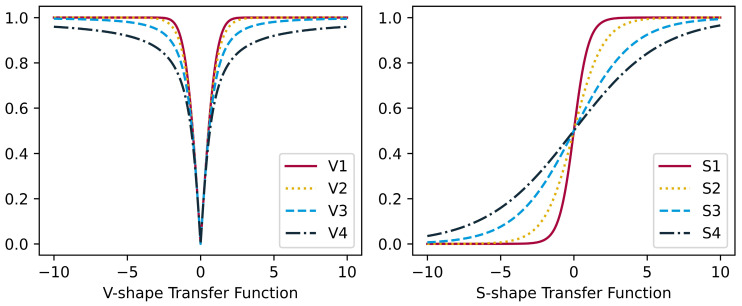
V-shaped and S-shaped transfer functions.

Metaheuristic algorithms inherently generate continuous solutions that lie within the upper and lower boundaries of the search space. One of the primary strategies to discretize these approaches is to utilize transfer functions. The primary objective of transfer functions is to acquire a 0–1 vector representing the characteristics to be chosen for feature selection while keeping the original procedure unchanged. The continuous values produced by the transfer function are transformed into either 0 or 1 using a thresholding operation. Thus, this approach yields a solution or possible answers to the binary optimization issue of identifying the most efficient characteristics for diagnosing respiratory disease. Transfer functions exhibit distinct characteristics, and when employed for discretization, varying outcomes are bound to arise [31]. Various transfer functions have been examined in many studies, but it remains necessary to make comparisons to determine which specific transfer functions should be employed to achieve discretization. To this end, this study utilizes a total of eight commonly employed transfer functions, which belong to two distinct families, namely S-shaped and V-shaped functions. Formulas for S-shaped transfer functions S1 to S4 and V-shaped transfer functions V1 to V4 are provided in Table 3. The function curves for them are given in Figure 1.

Our study introduces a wrapper feature selection strategy that utilizes the aforementioned metaheuristic search algorithms and the KNN classifier as the evaluator. When using feature selection methods, it is crucial to consider both a solution’s representation and the optimization process’s evaluation. The dataset used in this study exhibits a serious class imbalance issue. Thus, accurate prediction of minority classes is also crucial for a reliable model. Therefore, we used the Matthews correlation coefficient (MCC) as the fitness value for the optimization problem since the MCC metric is more suitable for distinguishing between various misclassification distributions in datasets with imbalanced class issues [32].

### 2.5. Classifier

When the studies in the field of feature selection using metaheuristic methods are analyzed, it is seen that the KNN algorithm has been frequently utilized as the classifier [33,34,35,36]. This is due to its simplicity and ease of application, as well as its ability to provide fairly accurate results. This is due to its simplicity, practicality, ease of application and use, as well as its ability to provide rapid and accurate results when dealing with large datasets. For the same reasons, a simple KNN (K = 5) classifier is utilized in this study. This classifier functions as a decision-maker during both the feature selection and classification phases. To make the comparisons fair, the same classifier was used in all trials.

## 3. Results

This study conducted experiments for comparing six different metaheuristics feature selection methods using eight different transfer functions from two families (V-shaped and S-shaped) to determine the best feature selection methods to be used for respiratory disease classification problems. This means finding the methods that lead to high classification performance while using a small number of features. Findings are examined, evaluated, and commented on below under three headings: transfer function fitness values, comparison of the classification performances of metaheuristic feature selection methods, and comparison with traditional feature selection methods.

### 3.1. Fitness Values of Transfer Functions for Each Feature Selection Method

**Figure 2:**
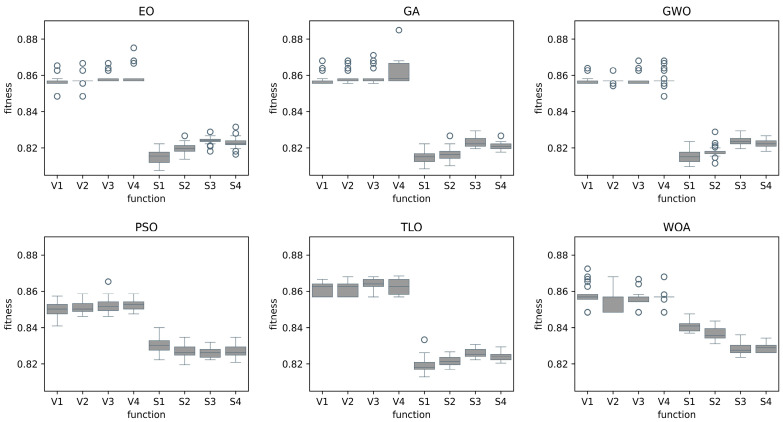
Fitness values of different transfer functions for each MHA FS method for Case 1.

**Figure 3:**
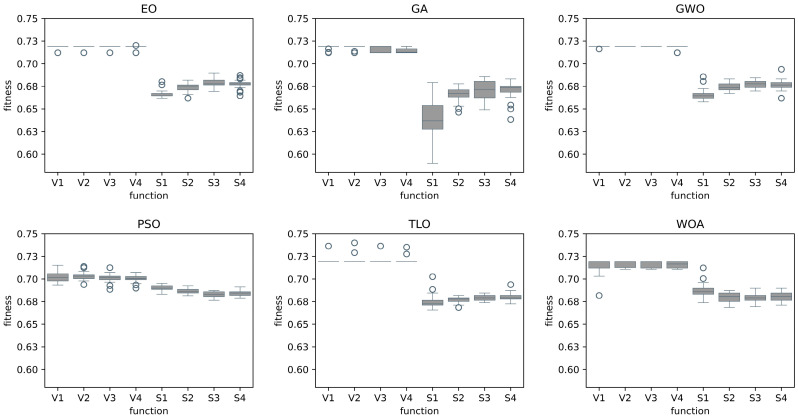
Fitness values of different transfer functions for each MHA FS method for Case 2.

During the feature selection phase, the classification model is evaluated using the test data, which is a randomly retained 20% of all data. The fitness values are computed by comparing the actual values with the predicted results. The fitness value is a numerical measure that quantifies the quality of a solution candidate with respect to the optimization problem at hand. In our study, fitness function takes into consideration both a classification performance metric, which is selected as MCC, and also the number of features selected. Therefore, it simultaneously tries to maximize MCC value while minimizing the number of features selected. This feature selection process is repeated 25 times for each transfer function for each optimization method. Since there is an issue of imbalance in the class labels for both Case 1 and Case 2, the MCC metric was used to utilize the results. Figure 2 and Figure 3 display the fitness values of eight transfer functions for each feature selection method obtained after 25 trials for Case 1 and Case 2, respectively.

The fitness values in Figure 2 and Figure 3 show the overall performance of the transfer functions. As can be seen in Section 3.3, there are significantly fewer number of features selected for V-shaped transfer functions, while MCC values are slightly higher for S-shaped transfer functions. Therefore, considering both factors, the fitness values of V-shaped functions are slightly higher. In addition, the vertical size of the plots, which represent the dispersion of fitness values in 25 trials, shows that V-shaped functions are generally more stable than S-shaped ones. This implies that the fitness values of V-shaped functions do not change much from trial to trial.

### 3.2. Comparison of Classification Performance of Metaheuristic Feature Selection Methods

Once the feature selection process was concluded, the KNN classifier was fed by the selected features for testing. The same test set used in the FS phase is also used for classification. However, whereas each sample is represented by 75 features in the FS phase, the number of features used for each sample in the classification stage is less than 75, depending on the transfer function used. Classification conditions are maintained consistent by using the same random state rate, which is chosen as 42, and identical test data for the evaluation of all methods. Evaluation of the classification results is made based on MCC scores since the dataset is highly imbalanced. Each metaheuristic method was executed 25 times for each of the eight different transfer functions (S1–S4, V1–V4). For each run, an MCC value was calculated, resulting in 200 MCC values per method. To compare the effects of the S and V-shaped transfer function families on classification performance we calculated the mean and standard deviation of 100 MCC scores obtained for both families for each metaheuristic method. The mean and standard deviation of MCC values for each transfer function family are presented in Table 4 for Case 1 and Table 5 for Case 2.

**Figure 4:**
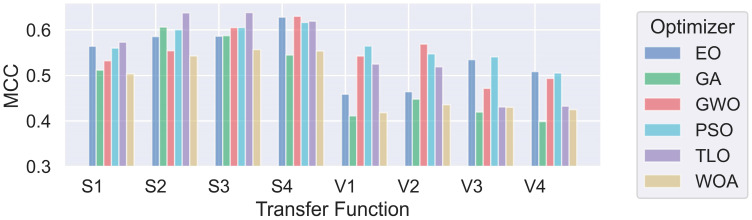
Comparison of MHA FS methods for individual transfer functions for Case 1.

**Figure 5:**
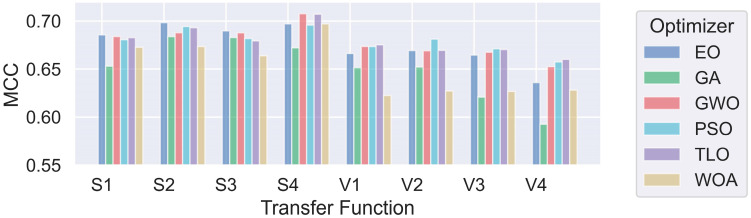
Comparison of MHA FS methods for individual transfer functions for Case 2.

The bold-styled values in these tables represent the best average scores obtained for each transfer function family. The performance results of each individual transfer function are also presented in a graphical format in Figure 4 and Figure 5. It can be observed that methods using S-shaped transfer functions obtain higher classification performance than those using V-shaped ones.

### 3.3. Comparison of Metaheuristic Feature Selection Methods Based on Number of Features Selected

In addition to classification performance, the number of features selected by metaheuristic algorithms for different transfer functions was also investigated and compared. Every metaheuristic method for each of the eight different transfer functions is employed 25 times and statistical averages (mean and standard deviation) are calculated for each of these 25 runs for fair assessment. We first compared the effects of transfer function families. In other words, we took the average of scores obtained for all V-shaped functions; we also did the same for all S-shaped functions. The results are presented in Table 6 and Table 7 for Case 1 and Case 2, respectively.

**Figure 6:**
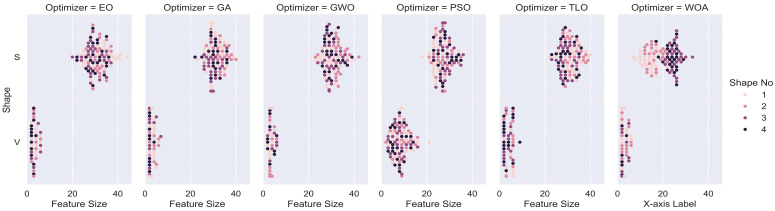
Selected feature sizes of MHA FS methods for S- and V-shaped transfer functions for Case 1.

**Figure 7:**
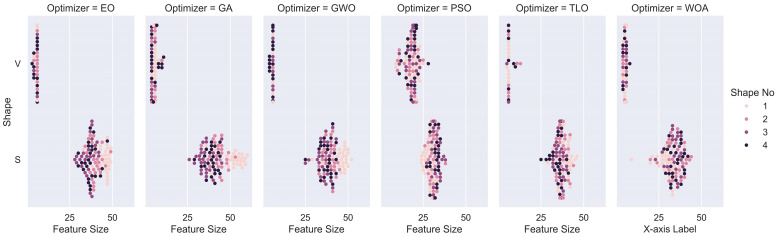
Selected feature sizes of MHA methods for S- and V-shaped transfer functions for Case 2.

The dispersion of selected feature size in one of 25 trials for each individual transfer function is also presented as a scatter plot in Figure 6 and Figure 7, for Case 1 and 2, respectively. It can be observed that methods using V-shaped functions use significantly fewer features than those using S-shaped ones.

### 3.4. Comparison of Metaheuristic Feature Selection Methods with Classical Feature Selection Methods

In this section, the effectiveness of metaheuristic feature selection methods is compared with the following classical feature selection methods: Spearman’s correlation (Spearman), mutual information (MI), relief, variance threshold (VAR), mean absolute difference (MAD), dispersion ratio (DR), lasso, tree-based, recursive feature elimination (RFE), and sequential forward selection (SFS). These classical methods were implemented using the Mafese library [37], which is an open-source Python library. The number of features to be selected was determined by ranking them based on their MCC scores. This was achieved by calculating all possible combinations of features within the range of minimum and maximum number of features for each method. The number of features that lead to the highest MCC value was taken as the optimum feature size for that method. The performance values are presented in Table 8 and Table 9 for Cases 1 and 2, respectively. The bold types represent the highest scores for each metric. In order to compare the results of classical feature selection methods with metaheuristic ones, the highest average performance values, obtained over 25 trials, for each metaheuristic method are presented in Table 10 and Table 11 for Cases 1 and 2, respectively. These tables also show with which transfer functions (one for S-shaped functions and the other for V-shaped functions), these values are obtained. Table 8, Table 9, Table 10 and Table 11 also represent the number of features selected by each method and their computation time.

## 4. Discussion

In this study, we explored and compared the performances of nature-inspired metaheuristic algorithms for feature selection in respiratory disease classification. The main objective is to enhance classification accuracy while reducing the feature set size, thereby improving the model’s computational efficiency. The experiments were conducted on a dataset consisting of respiratory sound recordings belonging to different diseases. Our approach utilized a variety of metaheuristic algorithms and two popular families of transfer functions to select the most relevant features. We first analyzed the fitness values of eight different transfer functions belonging to two families for each metaheuristic feature selection algorithm. It is seen that fitness values of V-shaped functions exhibit slightly higher values than S-shaped ones. In addition, the vertical size of the plots, which represent the dispersion of fitness values obtained in 25 trials (Figure 6 and Figure 7), shows that V-shaped functions are generally more stable than S-shaped ones. In other words, fitness values of V-shaped functions do not change much from trial to trial. Then we analyzed the effects of these MHA and transfer functions on the classification performance for two different cases. It reveals that MHA methods using S-shaped transfer functions obtain slightly better classification performance than the same methods when they use V-shaped functions. However, using V-shaped transfer functions has two important advantages. First, they are much more stable, which means their fitness values do not change much from trial to trial. Second, the number of features selected by MHA methods when employing V-shaped transfer functions is significantly less than the same methods when they use S-shaped functions.

Next, we compared the performances of MHA-based feature selection methods with classical feature selection methods based on MCC metric and number of features selected. Upon analysis of the results, it is seen that the proposed MHA-based feature selection methods obtain better results than most, if not all, of the classical methods in both binary and multi-class cases. The “Sequential” feature selection method, which is a classical one, obtained the highest scores in both cases. In addition, two other classical methods, “Spearman” and “Recursive,” obtained the second highest scores in Case 1 and Case 2, respectively. However, these methods select a large number of features, increasing the computational burden on the classification system. We also measured computational time of each method for the feature selection process. The MHA-based methods’ computational time is found to be significantly lower than FFS (Sequential) method. These results show that if, in addition to classification performance, small number of features and low computational time are required, then MHA feature selection methods (in particular, GWO and PSO) using V-shaped transfer functions should be chosen.

There is one point that needs explanation here. The accuracy values obtained by classical feature selection methods are higher than MHA-based methods. This result can lead to the conclusion that metaheuristic methods are worse. However, we want to emphasize that the Accuracy metric could be misleading in datasets with class imbalances. Therefore, in such datasets, metrics such as MCC, F1 or Balanced Accuracy should be used. Our study uses a highly imbalanced dataset. Thus, we used one of these metrics, i.e., MCC, for measuring classification performances. This way, we have derived models that can also accurately forecast minority classes.

A possible limitation of this study is that it was conducted using a single database. The results that would be obtained with other databases could be different than the ones obtained here. Another limitation is due to the database used since some disease classes are represented by very small numbers of data. Still another drawback is the use of only six MHA; although these are the most-used ones in the literature, there are other MHA that are not included in this study.

## 5. Conclusions

The main goal of this detailed comparison study is to examine the performance of different nature-inspired MHA for feature selection in respiratory disease classification. The results show that MHA-based feature selection algorithms provide robust mechanisms for navigating the high-dimensional feature space, ensuring that the selected features contribute maximally to the classification task. Furthermore, the reduction in the number of features, especially by using V-shaped transfer functions, not only decreases the computational load but also minimizes the risk of overfitting. This is particularly important in the context of respiratory disease classification, where the diversity and variability of the data can lead to complex decision boundaries. The overall improvement in classification accuracy and computational efficiency underscores the effectiveness of these algorithms in feature selection for respiratory disease classification. Future work could explore the combination of these algorithms with other advanced machine learning techniques, such as ensemble learning, to further enhance the robustness and generalizability of the models.

## Figures and Tables

**Table 1:** Feature extraction methods employed in the study.

No.	Method	Abbreviation	Explanation
1	Chroma Short Time Fourier Transform	Chro_stft	It calculates a chromatogram by analyzing either a waveform or a power spectrogram.
2	Chroma Constant-Q chromagram	Chro_cqt	It calculates the Constant-Q chromagram.
3	Chroma Energy Normalized	Chro_cens	It calculates the chroma variant known as Chroma Energy Normalized.
4	Chroma Variable-Q chromagram	Chro_vqt	It calculates the Variable-Q chromagram.
5	Mel-Frequency Cepstral Coefficients	Mfcc	It calculates the mel-frequency cepstral coefficients
6	Root Mean Square	Rms	It calculates the root mean square value for each frame, using either the audio samples or the spectrogram.
7	Spectral Centroid	Spec_cent	It calculates the spectral centroid. Every individual frame of a magnitude spectrogram is standardized and considered as a distribution across frequency bins. From this distribution, the average value is calculated for each frame.
8	Spectral Bandwidth	Spec_bandw	It calculates the spectral bandwidth.
9	Spectral Contrast	Spec_cont	It calculates the spectral content of each frame by dividing it into sub-bands. The energy contrast for each sub-band is determined by comparing the average energy in the highest quantile (peak energy) with that of the lowest quantile (valley energy).
10	Spectral Flatness	Spec_flat	It calculates the spectral flatness. Spectral flatness, also known as tonality coefficient, is a metric used to quantify the degree to which a sound resembles noise rather than a distinct tone. A spectral flatness value closer to 1.0 indicates that the spectrum is more like white noise.
11	Spectral Roll-off Frequency	Spec_rof	It calculates the roll-off frequency. The roll-off frequency is determined for each frame as the central frequency of a spectrogram bin that contains at least roll percent of the energy of the spectrum in that frame, as well as the bins below it.
12	Polynomial Features	Poly_fea	It obtains the coefficients for fitting a polynomial to the individual columns of a spectrogram.
13	Tonal Centroid Features	Tonnetz	It calculates the tonal centroid characteristics. This representation employs the technique of projecting chroma features onto a six-dimensional basis, where the perfect fifth, minor third, and major third are each represented by two-dimensional coordinates.
14	Zero Crossing Rate	Zero_cr	It calculates the rate at which the audio time series crosses the zero axis.

**Table 2:** Distribution of data in Case 1 and Case 2.

Case	Class	Sample Size	Number of Features
1	Healthy	35	75
	Diseased	885	
2	Healthy	35	
	Chronic diseased	810	
	Non-chronic diseased	75	

**Table 3:** V-shaped and S-shaped transfer functions.

V-Shape		S-Shape	
Name	Formula	Name	Formula
V1	$V1(x)=|erf(\frac{\sqrt{\pi }}{2}x)|$	S1	$S1(x)=\frac{1}{1+e^{-2x}}$
V2	V2(x)=|tanh(x)|	S2	$S2(x)=\frac{1}{1+e^{-x}}$
V3	$V3(x)=|\frac{x}{\sqrt{1+x^{2}}})|$	S3	$S3(x)=\frac{1}{1+e^{-(\frac{x}{2})}}$
V4	$V4(x)=|\frac{2}{\pi }arctan(\frac{\pi }{2}x)|$	S4	$S4(x)=\frac{1}{1+e^{-(\frac{x}{3})}}$

**Table 4:** Comparison of Metaheuristic Algorithms for S- and V-shaped Transfer Function Families for Case 1.

Shape	Method	Mean MCC	Std. D. of MCC
S	EO	0.5909	0.1211
	GA	0.5627	0.1206
	GWO	0.5803	0.1136
	PSO	0.5956	0.1043
	TLO	**0.6171**	0.1055
	WOA	0.5392	0.1174
V	EO	0.4916	0.1335
	GA	0.4193	0.1704
	GWO	0.5191	0.1476
	PSO	**0.5395**	0.1621
	TLO	0.4767	0.1891
	WOA	0.4272	0.1626

Numbers given in bold are the best scores of S and V shaped functions.

**Table 5:** Comparison of Metaheuristic Algorithms for S- and V-shaped Transfer Function Families for Case 2.

Shape	Method	Mean MCC	Std. D. of MCC
S	EO	**0.6927**	0.0528
	GA	0.6730	0.0548
	GWO	0.6918	0.0523
	PSO	0.6882	0.0414
	TLO	0.6905	0.0466
	WOA	0.6768	0.0550
V	EO	0.6590	0.0410
	GA	0.6292	0.0604
	GWO	0.6657	0.0345
	PSO	0.6709	0.0445
	TLO	**0.6688**	0.0326
	WOA	0.6262	0.0667

Numbers given in bold represents the best scores for S and V shapes in terms of Mean MCC metric

**Table 6:** Mean and standard deviation of number of selected features of MHA FS methods for Case 1.

Method	Shape	Mean	Std. D.	Shape	Mean	Std. D.
EO	S	30.90	4.49	V	3.21	0.94
GA		31.02	3.48		3.03	1.00
GWO		30.56	3.75		3.44	0.87
PSO		27.13	3.53		8.47	3.44
TLO		30.85	3.84		3.93	1.59
WOA		19.73	5.88		2.96	1.00

**Table 7:** Mean and standard deviation of number of selected features of MHA FS methods for Case 2.

Method	Shape	Mean	Std. D.	Shape	Mean	Std. D.
EO	S	39.57	5.29	V	5.83	0.59
GA		42.56	7.96		5.72	1.33
GWO		39.78	5.64		5.90	0.44
PSO		30.71	3.05		18.31	3.57
TLO		36.99	3.69		6.17	1.00
WOA		33.26	5.55		5.55	0.77

**Table 8:** Best performances of Classical FS methods for Case 1.

Method	ACC	F1 Macro	MCC	Number of Features	Comp. Time (s)
DR	0.978	0.744	0.506	17	1.21
Lasso	0.967	0.492	−0.012	3	1.24
MAD	0.973	0.493	0.000	7	2.77
MI	0.978	0.661	0.442	9	16.28
RELIEF	0.973	0.493	0.000	7	60.43
Recursive	0.978	0.744	0.506	31	758.31
SPEARMAN	**0.989**	0.872	0.770	55	2.68
Sequential	**0.989**	**0.897**	**0.794**	47	2597.45
Tree	0.978	0.661	0.442	11	6.26
VAR	0.973	0.493	0.000	7	0.10

Numbers given in bold represents the best values achieved for ACC, F1 Macro, and MCC metrics.

**Table 9:** Best performances of Classical FS methods for Case 2.

Method	ACC	F1 Macro	MCC	Number of Features	Comp. Time (s)
DR	0.918	0.689	0.597	18	1.46
Lasso	0.842	0.352	0.054	5	1.22
MAD	0.891	0.511	0.528	14	2.89
MI	0.891	0.511	0.528	57	17.35
RELIEF	0.891	0.511	0.528	14	58.73
Recursive	0.940	0.677	0.711	39	941.72
SPEARMAN	0.891	0.511	0.528	72	2.63
Sequential	**0.962**	**0.797**	**0.829**	67	2590.82
Tree	0.908	0.515	0.533	27	5.87
VAR	0.891	0.511	0.528	15	0.15

Numbers given in bold represents the best values achieved for ACC, F1 Macro, and MCC metrics.

**Table 10:** Best results according to the average of 25 trials of MHA FS methods for Case 1.

Method	Transfer Function	MCC	Balanced Accuracy	ACC	F1 Macro	Average Number of Features	Comp. Time (s)
EO	S4	0.628	0.650	0.918	0.630	29.92	150.98
	V3	0.535	0.613	0.905	0.612	3.36	108.22
GA	S2	0.606	0.631	0.914	0.616	33.44	152.08
	V2	0.448	0.564	0.891	0.567	3.20	102.35
GWO	S4	0.630	0.651	0.918	0.629	30.52	146.72
	V2	0.569	0.639	0.911	0.637	3.40	100.77
PSO	S4	0.616	0.658	0.916	**0.643**	28.08	150.78
	V1	0.564	0.619	0.908	0.610	9.24	140.44
TLO	S3	**0.638**	**0.659**	**0.920**	0.636	29.04	508.87
	V1	0.525	0.597	0.905	0.590	4.20	232.92
WOA	S3	0.557	0.611	0.903	0.599	25.56	285.29
	V2	0.436	0.538	0.888	0.536	3.16	124.85

Bold numbers represents the best value achieved for MM, Balanced Accuracy, ACC, and F1 Macro metrics.

**Table 11:** Best results according to average of 25 trials of MHA FS methods for Case 2.

Method	Transfer Function	MCC	Balanced Accuracy	ACC	F1 Macro	Average Number of Features	Comp. Time (s)
EO	S2	0.698	0.695	0.937	0.704	40.04	280.76
	V2	0.669	0.689	0.931	0.698	5.92	188.76
GA	S2	0.684	0.675	0.934	0.683	43.44	277.84
	V2	0.652	0.676	0.928	0.685	5.92	186.54
GWO	S4	**0.708**	**0.709**	**0.938**	**0.716**	37.6	151.40
	V1	0.674	0.696	0.931	0.706	6.00	128.04
**PSO**	**S4**	0.696	0.702	0.936	0.713	31.88	202.25
	**V2**	0.681	0.698	0.933	0.712	17.76	251.96
**TLO**	**S4**	0.707	0.703	**0.938**	0.710	34.68	342.42
	**V1**	0.675	0.694	0.931	0.701	5.96	423.54
**WOA**	**S4**	0.697	0.689	0.937	0.697	34.88	220.66
	**V4**	0.628	0.657	0.924	0.656	5.56	216.66

Bold numbers represents the best value achieved for MM, Balanced Accuracy, ACC, and F1 Macro metrics.

## Data Availability

The data used in this study are openly available in [ICBHI 2017] at [https://doi.org/10.1007/978-981-10-7419-6_6], reference number [28].
